# Encephalitic alphavirus infection induces long-term humoral and cell-mediated responses in seropositive participants from Panama

**DOI:** 10.1371/journal.pntd.0014660

**Published:** 2026-09-11

**Authors:** Courtney A. Cohen, Jean Paul Carrera, Josefrancisco Galué, Davis Beltran, Rita Corrales, Luis Felipe Rivera, Zumara Chaverra, Yelissa Suarez, Jennifer Marciaga, Michelle L. Rock, Megan C. Ha, Darrell L. Wetzel, Erick V. O’Brien, Dafna Abelson, Zachary A. Bornholdt, Crystal L. Moyer, Larry Zeitlin, Juan M. Pascale, John M. Dye, Sandra López-Vergés, Andrew S. Herbert

**Affiliations:** 1 U.S. Army Medical Research Institute of Infectious Diseases, Virology Division, Fort Detrick, Maryland, United States of America; 2 Department of Research in Virology and Biotechnology, Gorgas Memorial Institute for Health Studies, Panama City, Panama; 3 Regional Center for Innovation in Vaccines and Biopharmaceuticals (CRIVB AIP), Panama City, Panama; 4 Carson Centre for Health & Ecosystem Research, La Peñita, Darién, Panama; 5 Instituto de Investigaciones Científicas y Servicios de Alta Tecnología de Panamá (INDICASAT AIP), City of Knowledge, Panama City, Panama; 6 The Geneva Foundation, Tacoma, Washington, United States of America; 7 Oak Ridge Institute for Science and Education, Oak Ridge, Tennessee, United States of America; 8 Henry M. Jackson Foundation for the Advancement of Military Medicine, Bethesda, Maryland, United States of America; 9 Mapp Biopharmaceutical, Inc., San Diego, California, United States of America; 10 Eitr Biologics, Inc., San Diego, California, United States of America; 11 Clinical and Tropical Medicine Diagnostic and Research Unit, Gorgas Memorial Institute for Health Studies, Panama City, Panama; University of Tennessee College of Medicine: The University of Tennessee Health Science Center College of Medicine, UNITED STATES OF AMERICA

## Abstract

Encephalitic alphaviruses are an emerging viral threat with a history of large-scale outbreaks. Evaluating the immune response to natural infection in survivors is critical to the rational development of medical countermeasures (MCM), and yet little is known thus far. Here, we describe a comprehensive analysis of adaptive immune responses from a cohort of 37 convalescent seropositive individuals of Venezuelan equine encephalitis and/or Madariaga virus from Panama. We demonstrate persistent and polyfunctional B cell responses, as well as memory T cell cytokine production in response to viral antigen stimulation at least 3 years post-infection. VEEV survivors exhibit a higher proportion of strong responders with wider cross-reactivity than MADV survivors. This work illustrates the importance of a scientific focus on global health and international collaboration, explores several aspects of immunological engagement under-reported in the context of encephalitic alphaviruses, and should be leveraged to inform benchmarks for MCM evaluation and design.

## Author summary

The impacts of globalization and climate change continue to promote the emergence and spread of infectious viral diseases. Although encephalitic alphaviruses have caused significant human outbreaks for decades, there are no approved therapeutics or vaccines available for human use. Much work has described the impact of various immune responses on the pathology of and recovery from alphavirus infection in animals, and yet little is known about immune responses in humans. Here we describe persistent humoral and cell-mediated memory responses in a cohort of convalescent seropositive human participants from the Darién region of Panama, where pathogenic alphaviruses regularly circulate in rural agricultural communities. We did not identify a specific immunological signature of recovery, but rather a diverse range of memory responses that may be associated with age upon infection, time post-infection, severity of clinical symptoms or co-morbidities. The baseline assessment of immune profiles of alphavirus survivors presented here, and the established framework of international collaboration and cooperation, offers an opportunity for future studies focused on understanding how specific immune responses may influence severity and persistence of clinical symptoms or impact protection from co-circulating alphaviruses, as well as informing rational anti-VEEV and broader anti-neurotropic alphaviruses therapeutic and vaccine design through targeted immune engagement.

## Introduction

Alphaviruses from the *Togaviridae* family are composed of both encephalitic and arthritogenic varieties. Encephalitic strains include Venezuelan, Eastern and Western equine encephalitic viruses (VEEV, EEEV and WEEV), as well as Madariaga virus (MADV), formerly South American EEEV [1]. Although originally classified as Old World viruses, arthritogenic strains such as chikungunya virus (CHIKV), can also be found circulating in Central and South America, and others like Una virus (UNAV) and Mayaro virus (MAYV) are originally from the Americas. Alphaviruses are spherical, enveloped icosahedral virions, 70nm in diameter. The positive-sense RNA genome is encapsulated by the nucleocapsid, which is in turn surrounded by 80 trimeric spikes of the glycoprotein heterodimer (E1/E2) embedded into a lipid envelope [2–6]. E1 is positioned at the base of the trimeric spike, mediating low-pH endosomal fusion to facilitate release of the nucleocapsid into the infected cell cytoplasm [7]. E2 extends from the virion surface to engage with host cell surface receptors and mediate viral entry via receptor-mediated endocytosis [8,9]. Alphavirus E1 and E2 envelope glycoproteins are primary targets of the immune response, containing highly immunogenic sites that are targets of neutralizing antibodies.

Historically, VEEV has caused widespread outbreaks, at times infecting tens to hundreds of thousands of equids and humans in the Americas [10–12]. As a Category B pathogen that can rapidly replicate to high titer, has a low infectious dose, and is highly infectious by aerosol, VEEV is also a significant biodefense threat agent [13,14]. VEEV is composed of 6 antigenic subtypes (I-VI), the first being the most relevant to human health due to its outbreak history and potential. Some type I strains are considered epizootic (IA/B and IC) because they amplify to high titers in horses and are more pathogenic, while others (ID & IE) are considered enzootic, tending to circulate more readily in mosquitoes and rodents [15,16]. To date, the geographical distribution of these strains remains non-overlapping, perhaps due to naturally occurring ecological barriers [10,15–18].

Panama is an ideal site for the study of alphaviruses given its well-documented history of VEEV ID circulation, in addition to MADV and several arthritogenic alphavirus strains (UNAV, MAYV, CHIKV) [19]. Recent epidemiological data from villagers in the eastern province of Darién indicates that the seroprevalence of anti-VEEV ID is up to 75% of the population and positively correlates with age, indicating endemic transmission in the region [20,21]. In 2010, a simultaneous outbreak of co-circulating VEEV ID and MADV in Darien resulted in 99 total acute cases, 19 hospitalizations related to encephalitis, and one documented case of co-infection [19,21]. This was the first reported outbreak with mild and severe cases caused by MADV in humans; since then, statistical analysis of the 2017 Panama outbreak revealed an increase in endemic exposure in the Darién province over the last decade for both viruses [19,22]. Further, the number of infected individuals is assumed to be under-reported due to a lack of widespread diagnostic tools within the region [20].

The incubation period for encephalitic alphaviruses is fairly rapid (2–5 days), and is associated with acute febrile illness, vomiting and diarrhea, and sometimes severe neurological symptoms including convulsions, photophobia, confusion and somnolence [10,23,24]. Although persistent neurosequelae have been reported in 50–90% of EEEV survivors, there is no correlation with those that experienced confirmed encephalitis or severe neurological signs during acute infection, suggesting virus may persist in the immune-privileged central nervous system (CNS) even after mild clinical illnesses [21,24]. In Panama, while laboratory-confirmed cases of VEEV were mostly in adults and only ~10% experienced convulsions, MADV infection was much more common in children and convulsions were reported in 42% of pediatric cases [19,24,25].

Clearly, encephalitic alphaviruses represent an emerging threat with pandemic-level potential. However, no FDA-approved medical countermeasures (MCMs) have been developed to date [26,27], and infections are treated solely with supportive care. While a handful of anti-VEEV monoclonal antibodies have been evaluated in rodent and non-human primate models, none have advanced into clinical development, perhaps over concerns regarding rapidity of VEEV entry into the CNS space and subsequent inaccessibility to large antibody moieties that cannot cross the blood brain barrier (BBB) efficiently [28–41]. Domains A and B of E2 protein are the immunodominant targets of the neutralizing antibodies specific to VEEV or cross-reactive to alphaviruses, both murine (1A4A, 1A3B7, 3B2A-9, 3B4C-4) and human-derived (hVEEV-63) that have been evaluated in preclinical studies [28–38]. There is a live attenuated vaccine available to at-risk laboratory personnel, but due to high reactogenicity and the potential for adverse side effects, it is not approved for broader use [42–44]. Moreover, ongoing climate change and the expansion of suitable habitats for mosquito vectors and animal reservoirs highlight the likelihood of continued disease reemergence and the mounting importance of providing the global community with logically-designed and safe alphavirus MCMs to address this unmet public health need [26].

The evaluation of memory responses in convalescent human survivors or seropositive individuals of infectious diseases may be invaluable in defining the correlates of immunological protection that can be leveraged for rational vaccine design, or in providing immunological benchmarks for the evaluation of future MCMs [45, 46]. However, little is known about the memory responses that develop in individuals exposed to encephalitic alphaviruses. Given the prevalence of circulating encephalitic alphaviruses in Panama, and heightened importance of efficacious MCMs in the region, through international collaboration we established a cohort of 37 alphavirus survivors from Darién, Panama, that were confirmed seropositive for either VEEV, MADV or both (double-positive, DP). Here we present a comprehensive immunological profile of circulating humoral and cell-mediated memory responses amongst convalescent seropositive participants (SPPs). We aim to provide benchmarks for the evaluation of protective efficacy of future encephalitic alphavirus MCMs, guide future vaccine design, and establish ties with the impacted community to build a framework for future clinical trials.

## Methods

### Ethics statement

Alphavirus convalescent seropositive individuals were recruited through the Gorgas Memorial Institute for Health Studies (GMI) after obtaining informed consent. Participants in the current analysis were initially identified during seroprevalence studies from 2018-2019, approved by Gorgas Bioethical Research Committee (Gorgas-IRB) 096/CBI/ICGES/24. Follow-up samples of alphavirus convalescent survivors evaluated here were collected under the approval of Gorgas-IRB 176/CBI/ICGES/22. All participants provided written consent prior to enrollment in the study. All experiments were performed in accordance with ethical guidelines, relevant Human Use regulations and the Helsinki declaration.

### Cohort of encephalitic alphavirus survivors in Darien

During the 2010 VEEV and MADV outbreak the majority of cases were reported in the Aruza community in Darien, and followed up on in 2015 [21]. In 2018–2019 an alphavirus serosurvey at the community level was initiated, and a subset of participants positive for VEEV-, MADV- or DP-specific antibodies were invited to participate in the work described here. The cohort currently has 47 participants (Aza1-47) for longitudinal sampling, but enrollment continues and participation during each on-site collection is variable. Here, we describe our evaluation of samples gathered in 2022 (37 total SPPs); future work will describe longitudinal responses outside the scope of this manuscript. Cohort demographic information and reported symptoms are described in S4 Fig.

### Collection and processing of human samples

Whole blood samples were aspirated into sterile Serum Separator Tubes (SST, BD) and mononuclear Cell Preparation Tubes (CPT, BD) containing sodium citrate anticoagulant. SSTs were centrifuged at 1300xg for 10 minutes and sera was aliquoted and frozen at -80°C. CPTs were centrifuged at 1800xg for 15 minutes. Peripheral blood mononuclear cells (PBMCs) were collected, washed and aliquoted for immediate T cell stimulation assay use. Additional PBMC were collected and stored in liquid nitrogen.

### rE1/E2 proteins

All recombinant viral glycoproteins were expressed from stably transfected Schneider 2 (S2) insect cells. Constructs included an inducible metallothionein promoter and a C-terminal double Strep-Tag. Pools secreting each protein were scaled up in HyClone SFM4 insect media (Cytiva), induced with 0.5mM CuSO and expressed for 5 days in shake flasks at 120 RPM, 27˚C. Proteins were purified from clarified S2 culture supernatant via the C-terminal double strep-tag II sequence using affinity chromatography (5 ml. StrepTrap HP column, Cytiva). Monomer and trimer were separated on an S200Increase column (GE Healthcare) run in phosphate buffered saline. Constructs expressing envelope proteins from EEEV (FL91), VEEV IA/B (Trinidad Donkey), WEEV (CBA87), VEEV IC (V198), VEEV ID (3880), VEEV IE (Mena II) and MADV (PAN/247188/2010) included E3, E2 and E1 with a (G_4_S)_4_ linker replacing the 6K gene.

### End Titer ELISA

Microtiter plates were coated with alphavirus rE1/E2 (VEEV (IA/B, IC, ID, & IE), MADV, WEEV, CHIKV, UNAV), and incubated at 4°C overnight. Plates were blocked with 5% ChonBlock (Chondrex) for 1 hour at room temperature, and sera was serially diluted 3-fold beginning at 1:100 prior to addition to the wells. Following a 1 hour incubation, bound antibodies were detected using goat anti-human IgG conjugated with HRP (horseradish peroxidase) (Southern Biotech) and ABTS substrate (ThermoFisher). Absorbance was measured at 405nm using a spectrophotometer, and end titer was defined as the last dilution above a cut-off threshold defined as the average absorbance of control sera + 3x the standard deviation.

### Cell Lines

Vero-E6 (monkey, female origin, epithelial), THP-1 (human, male origin, monocyte) and HL-60 (human, female origin, myeloid) cells were obtained from the American Type Cell Culture Collection (ATCC). Vero-E6 cells were cultured in Minimal Essential Medium (MEM, ThermoFisher Scientific) supplemented with 10% heat-inactivated (HI) fetal bovine sera (FBS) and 1% penicillin-streptomycin at 5% CO_2_, 37°C. THP-1 cells were cultured in RPMI 1640 (Gibco) medium and supplemented as above, with the addition of 1% GlutaMax (Gibco). HL-60 cells were cultured in the presence of Iscove’s Modified Dulbecco’s Medium (IMDM, ATCC) supplemented with 20% FBS, 1% L-glutamine (Corning) and 1% penicillin-streptomycin. HL-60s were differentiated into mature polymorphonuclear cells by supplementing media with 1.3% DMSO for 5 days at 37°C [47].

### Authentic virus microneutralization assays

Sera was serially diluted beginning at 1:100 and pre-incubated with alphavirus isolates for one hour, in triplicate. Strains were as follows: VEEV IA/B (Trinidad Donkey), VEEV IC (V198), VEEV ID (3880), VEEV IE (Mena II), MADV (Pan247188), EEEV NA (FL93), WEEV (CBA87), CHIKV (AF15561). Virus:sera inoculums were then added to Vero E6 cell monolayers for an additional 17 hours at 37°C. Cells were formalin-fixed and sequentially stained with an anti-virus mAb (VEEV IA/B, IC and ID: 1A4A, VEEV IE: 1A3B7, MADV and EEEV: mAb8754 from Sigma, WEEV: 9F12, CHIKV: 5G11), a goat anti-mouse IgG alexafluor488-conjugated secondary antibody, and Hoescht stain. All VEEV and WEEV detection mAbs were produced from hybridomas by USAMRIID’s Cell Culture Division. CHIKV detection antibody was a kind donation from Dr. J. Lai (Albert Einstein College of Medicine). Data was acquired on a Cytation. Percent viral inhibition was determined in comparison to infected, untreated Vero cells in an automated fashion with BioTek Gen5 software, a dose-response curve was generated, and sera dilution at which 50% of virus is neutralized (NT_50_) in comparison to control-infected wells is reported.

### Effector function assays

Antibody-dependent cellular phagocytosis (ADCP), antibody-dependent neutrophil phagocytosis (ADNP) and antibody-dependent complement deposition (ADCD) were evaluated as previously described [48–51]. Briefly, ADCP utilized rE1/E2 antigens (Mapp Bio) that were biotinylated as directed using NHS-LC-LC biotin (ThermoFisher) and coupled to yellow-green Neutravidin beads (Life Technologies). Sera was diluted 100-fold from 1 x 10^2^ – 1 x 10^6^ in culture media in duplicate and incubated with rE1/E2-coated beads for 2 hours at 37°C. Unbound antibodies were removed via centrifugation and THP-1 cells were added at 2.5 x 10^4^ cells per well. Cells were paraformaldehyde-fixed and analyzed by flow cytometry. A phagocytosis score was determined using the following formula: (percentage FITC^+^ cells)*(geometric mean fluorescent intensity of FITC^+^ cells)/10,000. ADNP also utilized biotinylated, coupled rE1/E2 incubated in the presence of diluted sera (as above) for 2 hours at 37°C. Differentiated HL-60 cells were added at 5 x 10^4^ cells per well, and incubated for 1 hour, 37°C. Cells were stained for CD11b (BD Biosciences), paraformaldehyde-fixed and analyzed by flow cytometry. A phagocytic score was determined as described for ADCP within the CD11b^+^ population. To measure ADCD, biotinylated rE1/E2 was coupled to red Neutravidin beads (ThermoFisher) and incubated with duplicate sera samples for 2 hours at 37°C to form immune complexes. After washing, lyophilized guinea pig complement (Cedarlane) diluted in gelatin veronal buffer (Sigma-Aldritch) was added for 30 minutes, followed by incubation with FITC-conjugated guinea pig complement C3 (MP Biomedical) for 15 minutes. Complement deposition was analyzed by flow cytometry, and the median C3 fluorescence was reported.

### Flow cytometry

Freshly harvested PBMCs were stimulated *ex vivo* in culture media (RPMI containing 5% heat-inactivated fetal bovine serum) containing 0.3µM VEEV (IA/B) or MADV rE1/E2 in polypropylene 5ml culture tubes (Falcon). For ex vivo stimulation, VEEV (IA/B) was chosen based on antigen availability at the time of the assay. To maximize available material for this analysis, the number of input cells is not standardized across donors. After 2 hours of incubation at 37°C, monensin was added (1 µM), and the cells were incubated for an additional 16 hours at 37°C. Cells were washed, plated in round-bottom 96-well plates and stained for dead cell exclusion (Zombie Aqua fixable live/dead stain), and T cell-specific surface markers (CD3, CD4, CD8, CD95, CD45RA, CCR7). Cells were then fixed, permeabilized and stained for intracellular cytokines (IFNγ, IL-2). Acquisition was performed on a Fortessa X20 (BD Biosciences) and data was analyzed using Flowjo software (TreeStar). Gating strategy was performed as described previously [46]. Depending on the density of cells in a sample, acquisition rates were optimized to efficiently process samples while maintaining an abort rate of <1%; all samples were fully acquired to maximize data yields.

### Statistical analysis

Longitudinal ELISA and microneutralization data was evaluated via linear mixed effects model, multiplicity was adjusted by Dunnett’s method; ns > 0.05. T cell cytokine responses were compared via pairwise linear mixed effects model. Significance described as *: p < 0.05, **: p < 0.01, ***: p < 0.001, ****: p < 0.0001. Statistical analysis was carried out using SAS version 9.4 (SAS Institute Inc., Carry NC).

### Data availability statement

Data available upon request. Due to increased security concerns associated with federally-controlled BSAT (biological select agent and toxins), data cannot be posted to a public forum. A data transfer agreement may be initiated upon request to Dr. Andrew Herbert, corresponding author, and re-use of this data will be subject to details specified in the agreement.

## Results

### Cohort breakdown

During seroprevalence studies performed by the GMI in Darién, 65 participants were recruited in 2015 and 47 between 2018 and 2019, of which a total of 95 (48 from the first study and all from the second study) were seropositive. This work describes enrollees (“Azas”) that were previously identified as either VEEV^+^, MADV^+^ or VEEV^+^MADV^+^ double positive (DP) based on serology (IgM+ or IgG+) and PRNT [19,21], and negative for additional circulating alphaviruses (CHIKV, UNAV, MAYV). Seropositive participants (SPPs) were infected before the serosurveys, and the samples described here were collected in 2022, indicating a post-infection interval of at least three years.

### ELISAs, cross-reactivity

**Fig 1:**
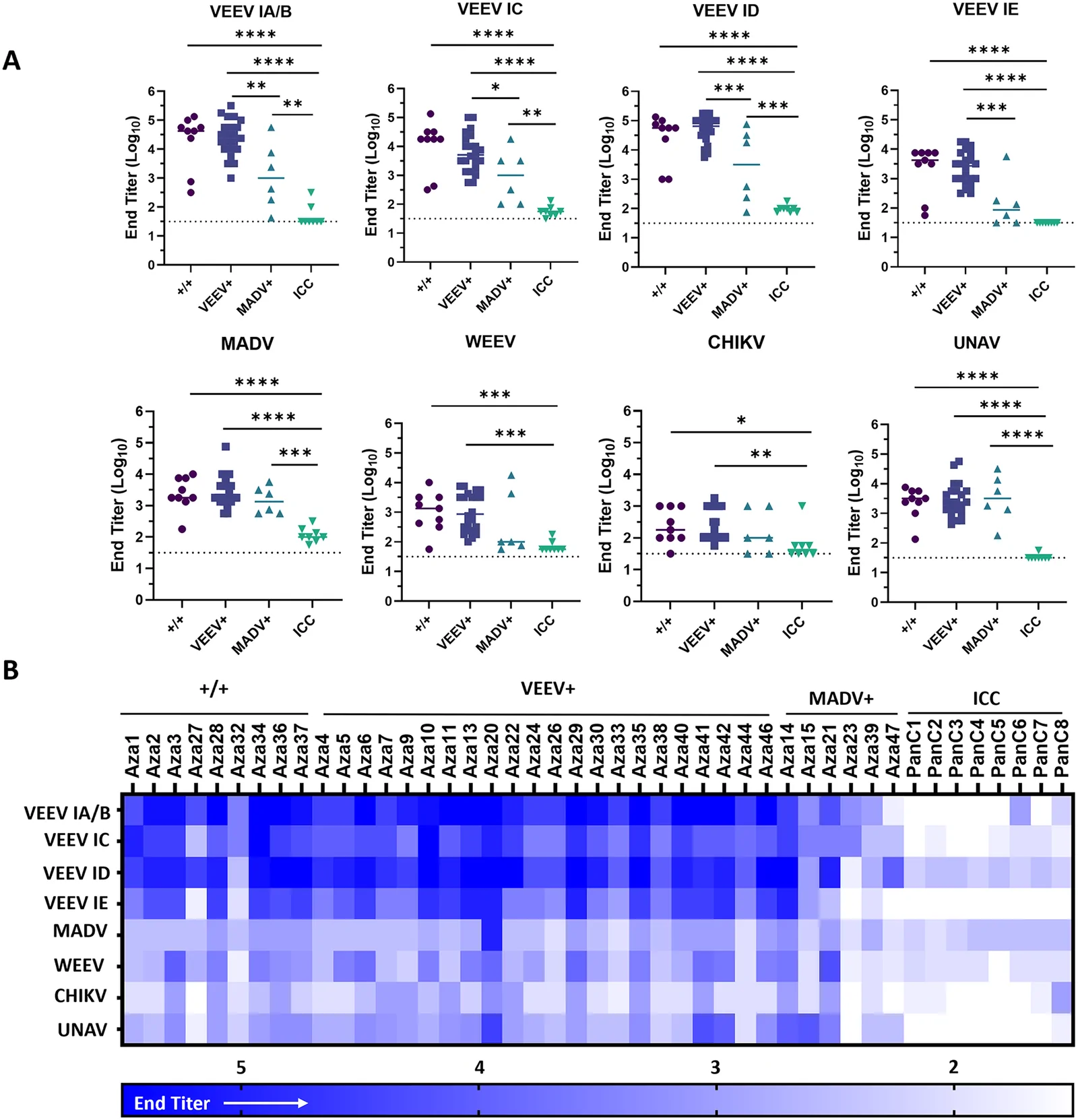
Convalescent SPP sera reactivity against alphavirus recombinant E1/E2. Sera samples were evaluated by end titer ELISA (A,B) for IgG binding to recombinant E1/E2 antigens to demonstrate broad reactivity. Dotted line (A) indicates assay limit of detection. “+/+” indicates DP individuals. Data represents two replicate assays. All pairwise comparisons were made against ICC and MADV+ cohorts, only those that reached significance are indicated. Significance described as *: p < 0.05, **: p < 0.01, ***: p < 0.001, ****: p < 0.0001.

To evaluate humoral responses, end-titer ELISAs were developed against rE1/E2 of several VEEV subtypes (IA/B, IC, ID, & IE), as well as additional encephalitic (MADV, WEEV) and arthritogenic (CHIKV, UNAV) alphaviruses. As shown in Fig 1A, VEEV^+^ participants demonstrated significant reactivity to all VEEV subtypes tested in comparison to in-country controls (ICC), representing uninfected bystander samples (p < 0.0001). The DP cohort were similarly reactive across VEEV subtypes (p < 0.0001) and even MADV^+^ individuals demonstrated significant cross-reactivity to all but the most divergent VEEV subtype (IE) in comparison to ICCs (p < 0.01 – 0.001). Although EEEV rE1/E2 was not available, all three cohorts were significantly seroreactive against MADV rE1/E2 (p < 0.001 – 0.0001). VEEV^+^ and DP cohorts also demonstrated significant reactivity against WEEV (p < 0.0001), and CHIKV (p < 0.05 – 0.01) in comparison to ICCs, and all three SPP cohorts demonstrated significant reactivity against UNAV (p < 0.0001). By breaking data out by individual (Fig 1B), we identified SPPs with high cross-reactive antibody titers by ELISA, predominantly from the DP and VEEV^+^ cohort (i.e., Aza20). We also demonstrate the broad, persistent humoral immunity in this cohort >3 years post-infection.

### Neutralizing antibodies

**Fig 2:**
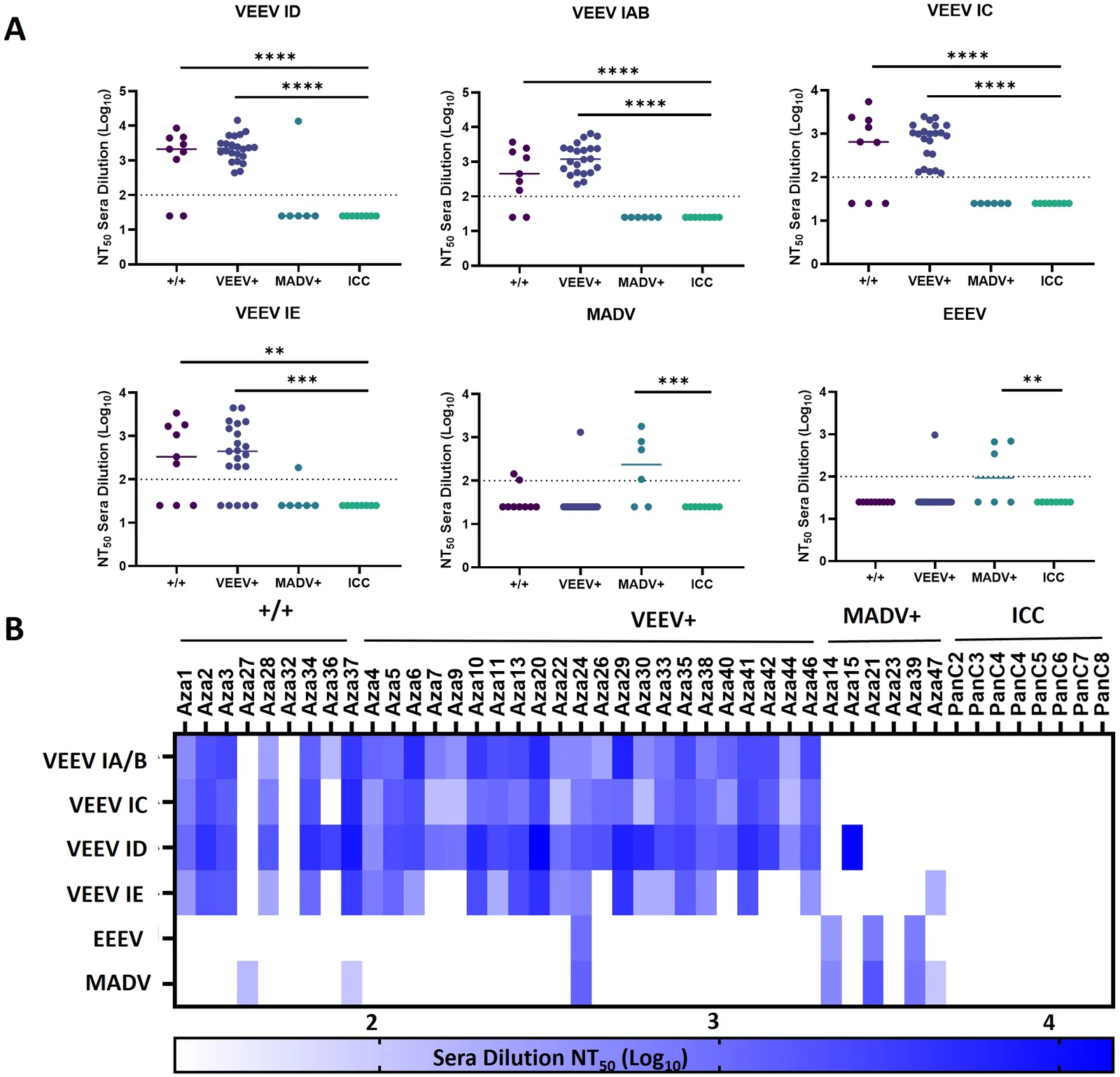
Convalescent SPP sera neutralization against authentic alphaviruses. Sera samples were evaluated by microneutralization assay (A,B) for reduction of Vero cell infection with VEEV subtypes IA/B (Trinidad Donkey strain), IC (P676 strain), ID (3880 strain), IE (Mena II strain), EEEV (FL93 strain) and MADV (Pan247188 strain). “+/+” indicates DP individuals. Data represents the sera dilution at which 50% of virus infection was neutralized (NT_50_), in comparison to untreated control wells. The dotted line represents the highest sera dilution used in the assay (1:100), and those samples that demonstrated no viral neutralization were assigned an “NT_50_” of 1:25 to demonstrate that while samples were tested, there was no detectable response in this assay. Data reflects 2-3 replicate assays performed in triplicate. All pairwise comparisons were made against ICC and MADV+ cohorts, only those that reached significance are indicated. Significance described as *: p < 0.05, **: p < 0.01, ***: p < 0.001, ****: p < 0.0001.

To determine neutralization activity, sera samples were serially diluted and incubated with authentic alphaviruses. As shown in Fig 2A, these data have been summarized as the 50% neutralizing titer (NT_50_) for ease of reporting (individual curves can be found in S1 Fig). All VEEV^+^ and most DP individuals demonstrate significant neutralization against “homologous” (regionally circulating) VEEV ID (3880 strain) compared to ICCs (p < 0.0001), while MADV^+^ participants were unresponsive. Further, VEEV^+^ and DP individuals also demonstrated significant cross-neutralization activity against VEEV IA/B (Trinidad Donkey strain, p < 0.0001), VEEV IC (V198 strain, p < 0.0001), and VEEV IE (Mena II strain, p < 0.001 and p < 0.01, respectively for VEEV^+^ and DP individuals) compared to ICCs. While some individuals demonstrated more cross-reactive circulating anti-VEEV antibodies overall (Fig 2B) (i.e. Aza37, Aza20 & Aza35), others had limited anti-VEEV IE titers (i.e. Aza26, Aza40 & Aza42), which is unsurprising as it is the most divergent Subtype I strain evaluated. Of interest, a single MADV+ individual (Aza15) demonstrated neutralization against VEEV ID, suggesting a potential secondary exposure.

Conversely, MADV^+^ participants were the only cohort able to significantly neutralize MADV (PAN247188 strain, p < 0.001) and EEEV (FL93 strain, p < 0.01) in comparison to ICCs. Of note, one individual in the VEEV^+^ cohort was also able to neutralize EEEV and MADV (Aza24), which again may be indicative of a subsequent exposure following sero-surveillance and cohort assignment. Although we also evaluated cohorts for broader cross-neutralization (WEEV & CHIKV), only one participant was able to neutralize CHIKV, also suggesting a secondary exposure (Aza22) (S1 Fig).

### Effector functions

**Fig 3:**
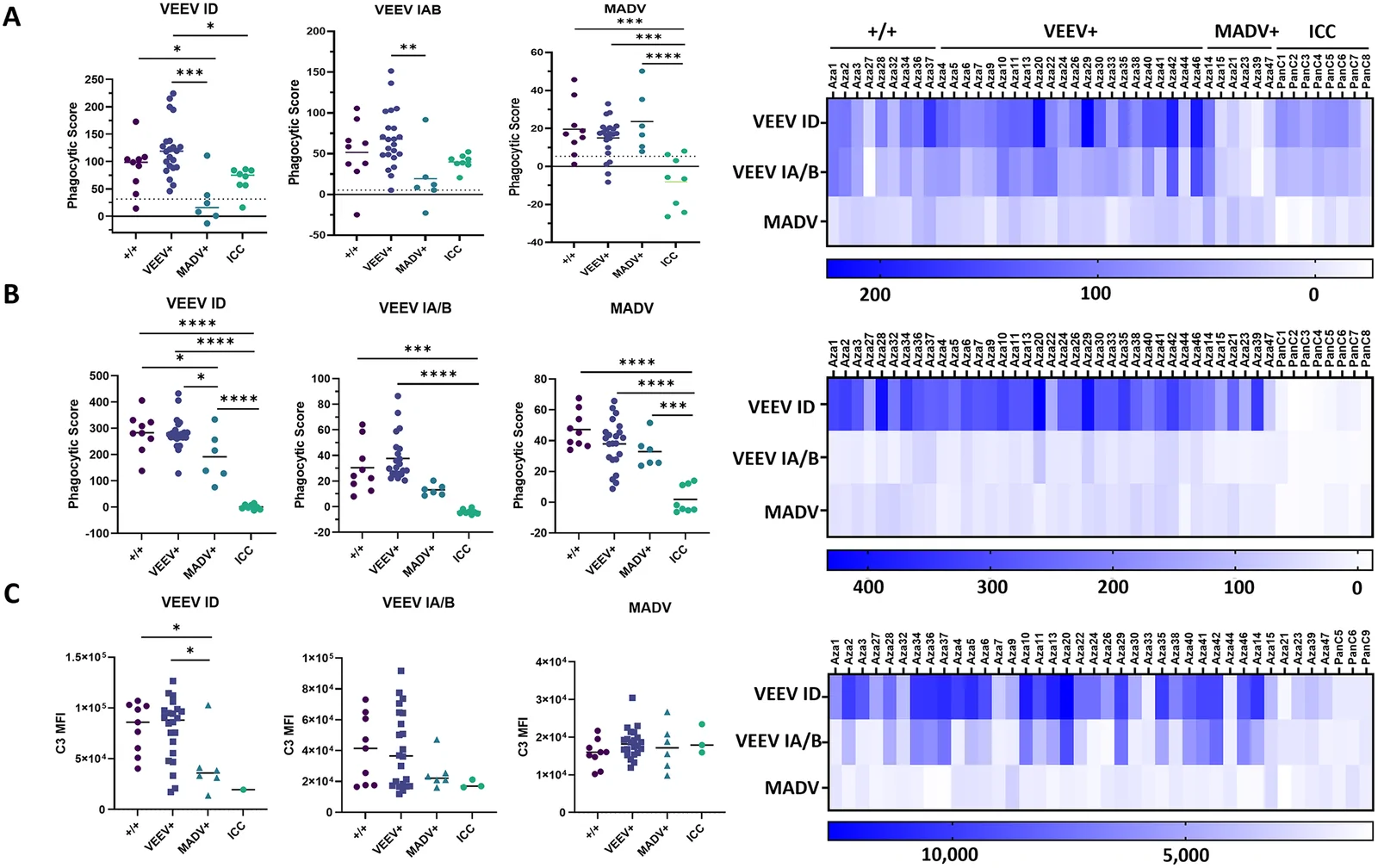
Convalescent SPP sera effector function. Longitudinal sera samples were evaluated for **(A)** ADCP, **(B)** ADNP and **(C)** ADCD activity against VEEV ID rE1/E2, VEEV IAB rE1/E1 and MADV rE1/E2. ADCP and ADNP phagocytic scores were normalized against cells that received conjugated beads without sera. ADCD is reported as the MFI of FITC signal. All pairwise comparisons were made against ICC and MADV+ cohorts, only those that reached significance are indicated. Significance described as *: p < 0.05, **: p < 0.01, ***: p < 0.001, ****: p < 0.0001.

It has recently been demonstrated that antibody effector functions are an important aspect of mAb-associated alphavirus protection [28]. Antibody effector functions are mediated via interaction between the Fc region of the antibody and Fc gamma receptors present on various immune effector cells, or directly with components of the complement system to facilitate clearance of virus or virally infected cells [52,53]. We evaluated the ability of circulating antibodies to mediate effector functions, specifically ADCP, ADNP and ADCD against VEEV and MADV rE1/E2-coated targets. As shown in Fig 3A, VEEV^+^ individuals demonstrated significantly elevated ADCP activity against VEEV ID when compared to ICC controls (p < 0.05). Further, both DP and VEEV^+^ participants demonstrated significantly higher ADCP activity against VEEV ID as compared to MADV+ participants (p < 0.05 and p < 0.001, respectively). VEEV^+^ participants also demonstrated significantly elevated ADCP activity against VEEV IA/B in comparison to MADV^+^ participants (p < 0.01). Conversely, all SPPs demonstrated significant ADCP activity against MADV as compared to ICCs (p < 0.0001).

Polyclonal SPP sera also demonstrated potent ADNP activity. As shown in Fig 3B, all SPPs demonstrated significantly elevated ADNP activity against VEEV ID, as compared to ICCs (p < 0.0001), although VEEV^+^ participants were significantly more reactive than MADV^+^ participants (p < 0.05). Anti-VEEV IA/B responses in DP and VEEV^+^ cohorts are similar, albeit less robust in scale. All SPP cohorts also demonstrate significant ADNP against MADV rE1/E2 as compared to bystander ICCs (p < 0.001 – 0.0001).

Finally, SPP and ICC sera were evaluated for ability to induce C3 deposition onto rE1/E2-coated beads, to represent complement-mediated killing of antigen-expressing cells in the context of an infection. Sera from DP and VEEV^+^ cohorts was significantly reactive against VEEV ID (p < 0.05), but not VEEV IA/B, in comparison to MADV^+^ participants. It is also interesting to note that while anti-VEEV ID induced a polyfunctional humoral response (ADCP, ADNP and ADCD) in the VEEV^+^ cohort, MADV rE1/E2 did not induce a robust ADCD response in the MADV^+^ cohort.

### T cell responses

In order to evaluate cell-mediated responses, PBMCs were obtained for antigen-specific T cell analysis by flow cytometry. Due to limitations in stimulating antigen and on-site logistics in Darien, only 13 individuals were selected for T cell analysis based on timing of sample acquisition in the field. We stimulated convalescent PBMCs overnight with either VEEV or MADV rE1/E2 and monensin, and stained for live/dead exclusion, conventional T cell markers, as well as phenotypic and activation markers to detect antigen-specific cytokine responses and to identify antigen-specific memory cell types. Memory subtypes were defined as either effector memory (T_EM_, CD95^+^CD45RA^-^CCR7^-^), central memory (T_CM_, CD95^+^CD45RA^-^CCR7^+^) or terminally differentiated memory (T_EMRA_, CD95^lo^CD45RA^+^CCR7^-^).

### CD4 responses

Here, we report antigen-specific memory CD4^+^ T cell SPP responses to VEEV and MADV rE1/E2. For simplicity, we are describing percent of cytokine-producing (IFNγ+ and/or IL-2+) cells representing a > 3-fold increase from the average negative stimulation as “potent” in the sections below which focus on CD4 T memory cell subsets. Representative scatter plots for T_CM_ populations can be found in S2 Fig Material.

**Fig 4:**
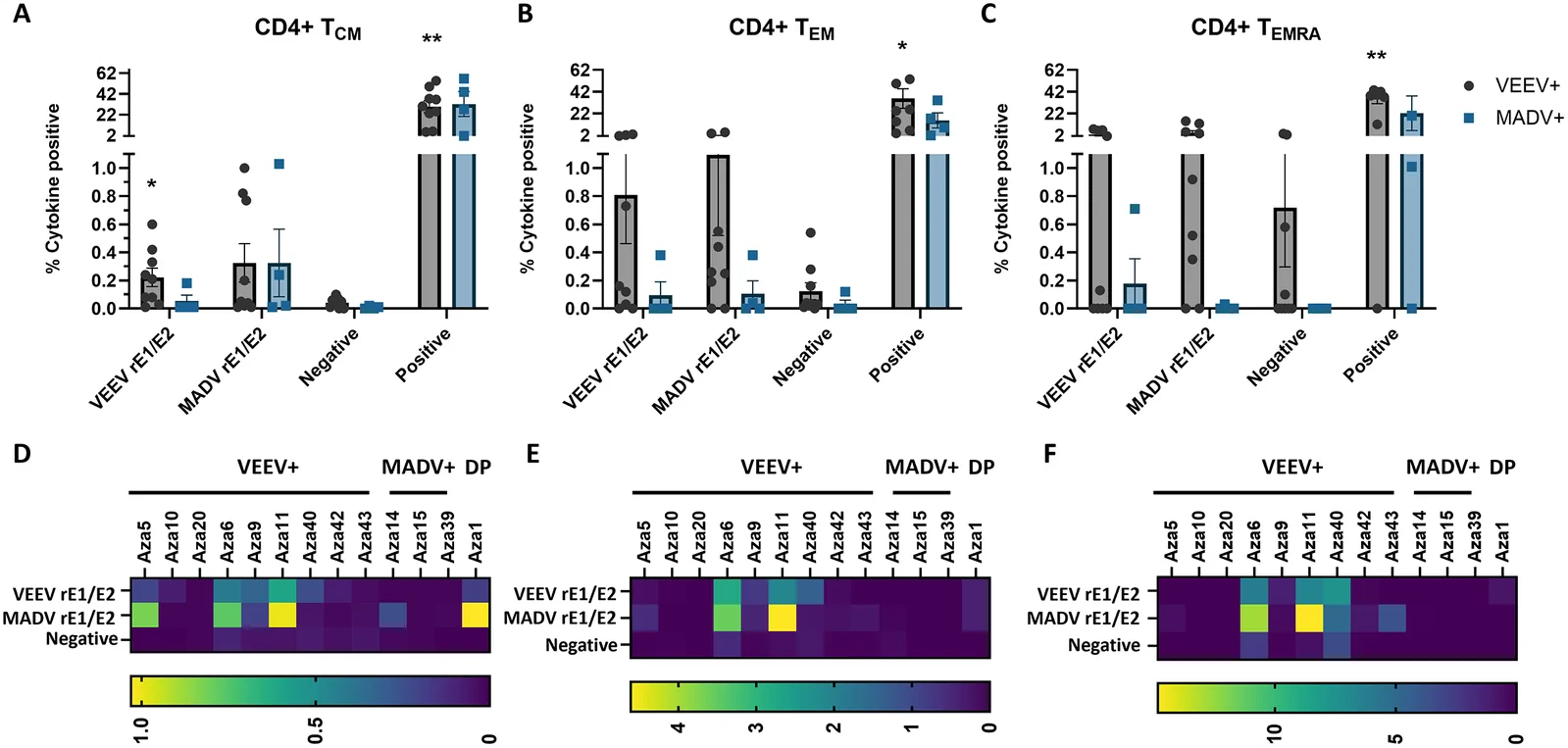
Convalescent SPP alphavirus-specific CD4^+^ T cell responses. PBMCs were stimulated for 18 hours with VEEV rE1/E2, MADV rE1/E2, media alone (“Negative”), or PMA/ionomycin (“Positive”), stained for dead cell exclusion, conventional T cell markers and intracellular cytokines, and analyzed by flow cytometry. Total cytokine production (IFNy + , IL-2+ and IFNy + IL-2+) in **(A)** T_CM_ (CD4^+^CD45RA^-^CD95^+^CCR7^+^), **(B)** T_EM_ (CD4^+^CD45RA^-^CD95^+^CCR7^-^) and **(C)** T_EMRA_ (CD4^+^CD45RA^+^CD95^lo^CCR7^-^) populations displayed by individual within the VEEV^+^ (black) and MADV^+^ (blue) cohorts. Single DP sample was combined with MADV^+^ survivors for simplicity **(A-C)**. Antigen-specific responses were compared to negative stimulation via pairwise linear mixed effects model. Significance described as *: p < 0.05, **: p < 0.01, ***: p < 0.001. Individual reactivity against VEEV rE1/E2, MADV rE1/E2 and media alone was broken out into heat maps in CD4^+^ **(D)** T_CM_, **(E)** T_EM_, and **(F)** T_EMRA_ populations to identify universal high responders and those with a marked response in a particular CD4^+^ subset. Arrows indicate maximum baseline response amongst individuals following media-only stimulation. Positive stimulation was removed from these graphs to improve scale resolution. Data represents a single assay on fresh cells.

Overall, 5/9 VEEV^+^ and the single DP sample demonstrated potent cytokine production in response to VEEV rE1/E2 (p < 0.05 in comparison to negative stimulation), while 4/9 VEEV^+^, 1/3 MADV^+^ and the DP participants had potent cytokine production in response to MADV rE1/E2 in the CD4^+^ TCM compartment (Fig 4A). In the CD4^+^ TEM compartment, 4/9 VEEV participants and the DP individual demonstrated potent cytokine production to both VEEV and MADV rE1/E2 (Fig 4B). In the CD4^+^ TEMRA compartment, 3/9 VEEV participants produced potent cytokine responses to VEEV rE1/E2, and 4/9 VEEV participants in response to MADV rE1/E2. To evaluate whether cytokine responses were indicative of overall high and low responders, we’ve also broken data out by individual (Fig 4D-4F). While some SPPs demonstrate cytokine production in all CD4^+^ memory T cell subsets (i.e., Aza11, Aza6), others produce cytokines in a limited subset of memory CD4^+^ cells (Aza43 against MADV rE1/E2 in CD4^+^ T_EMRA_, Aza5 and Aza1 against MADV in CD4^+^ T_CM_). It is also important to note that some SPPs had undetectable CD4^+^ T cell responses to alphavirus antigen stimulation (i.e., Aza20, Aza15, Aza39).

### CD8 responses

**Fig 5:**
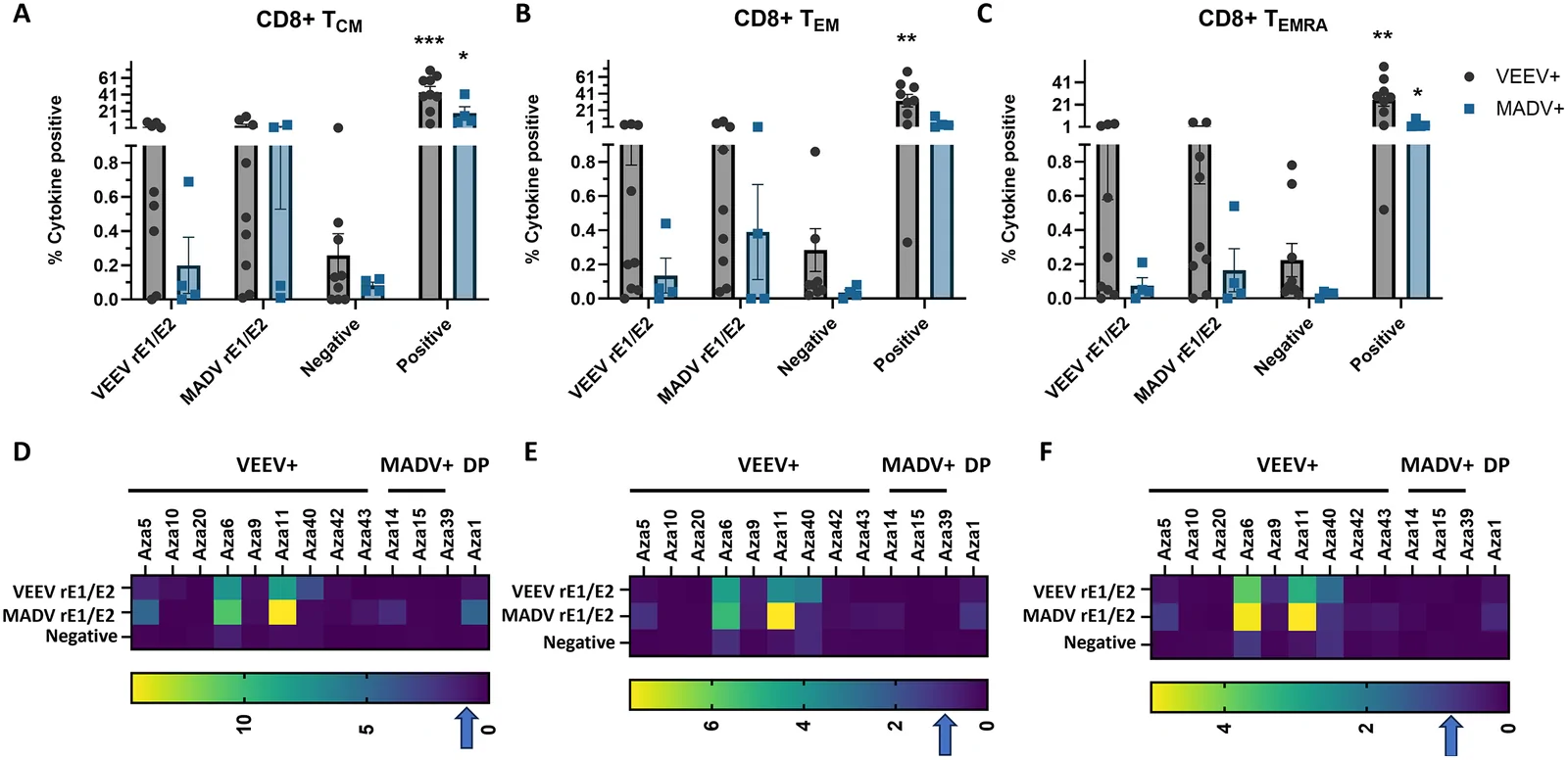
Convalescent SPP alphavirus-specific CD8^+^ T cell responses. PBMCs were stimulated for 18 hours with VEEV rE1/E2, MADV rE1/E2, media alone (“Negative”), or PMA/ionomycin (“Positive”), stained for dead cell exclusion, conventional T cell markers and intracellular cytokines, and analyzed by flow cytometry. Total cytokine production (IFNy^+^, IL-2^+^ and IFNy^+^IL-2^+^) in **(A)** T_CM_ (CD4^+^CD45RA^-^CD95^+^CCR7^+^), **(B)** T_EM_ (CD4^+^CD45RA^-^CD95^+^CCR7^-^) and **(C)** T_EMRA_ (CD4^+^CD45RA^+^CD95^lo^CCR7^-^) populations displayed by individual. Single DP sample was combined with MADV^+^ survivors for simplicity **(A-C)**. Individual reactivity against VEEV rE1/E2, MADV rE1/E2 and media alone was broken out into heat maps in CD8^+^ **(D)** T_CM_, **(E)** T_EM_, and **(F)** T_EMRA_ populations to identify universal high responders and those with a marked response in a particular CD8^+^ subset. Positive stimulation was removed from these graphs to improve scale resolution. Arrows indicate maximum baseline response amongst individuals following media-only stimulation. Data represents a single assay on fresh cells.

We also demonstrated potent (>3-fold increase over average negative stimulation) antigen-specific memory CD8^+^ T cell responses in alphavirus SPPs against VEEV and MADV rE1/E2. Overall, 5/9 VEEV^+^ and the DP participants demonstrated potent cytokine production in response to VEEV rE1/E2, while 4/9 VEEV^+^, 1/3 MADV^+^ and the DP participant had potent cytokine production in response to MADV rE1/E2 in the CD8^+^ T_CM_ compartment (Fig 5A). In the CD8^+^ T_EM_ and T_EMRA_ compartments, 4/9 VEEV^+^ participants demonstrated potent cytokine production to both VEEV and MADV rE1/E2, while the DP participant was reactive to MADV rE1/E2 only (Fig 5B-5C). As above, some SPPs (i.e., Aza11, 6) were consistently high responders across both CD4^+^ and CD8^+^ T cell subsets, while others produced cytokines in a more limited memory T cell subset (i.e., Aza5 and Aza1 against MADV rE1/E2 in CD8^+^T_CM_) (Fig 5D-5F). Taken together, this data demonstrates that VEEV infection may provide some anti-MADV T cell cross-reactivity, but the reverse was not noted in this small sampling.

**Fig 6:**
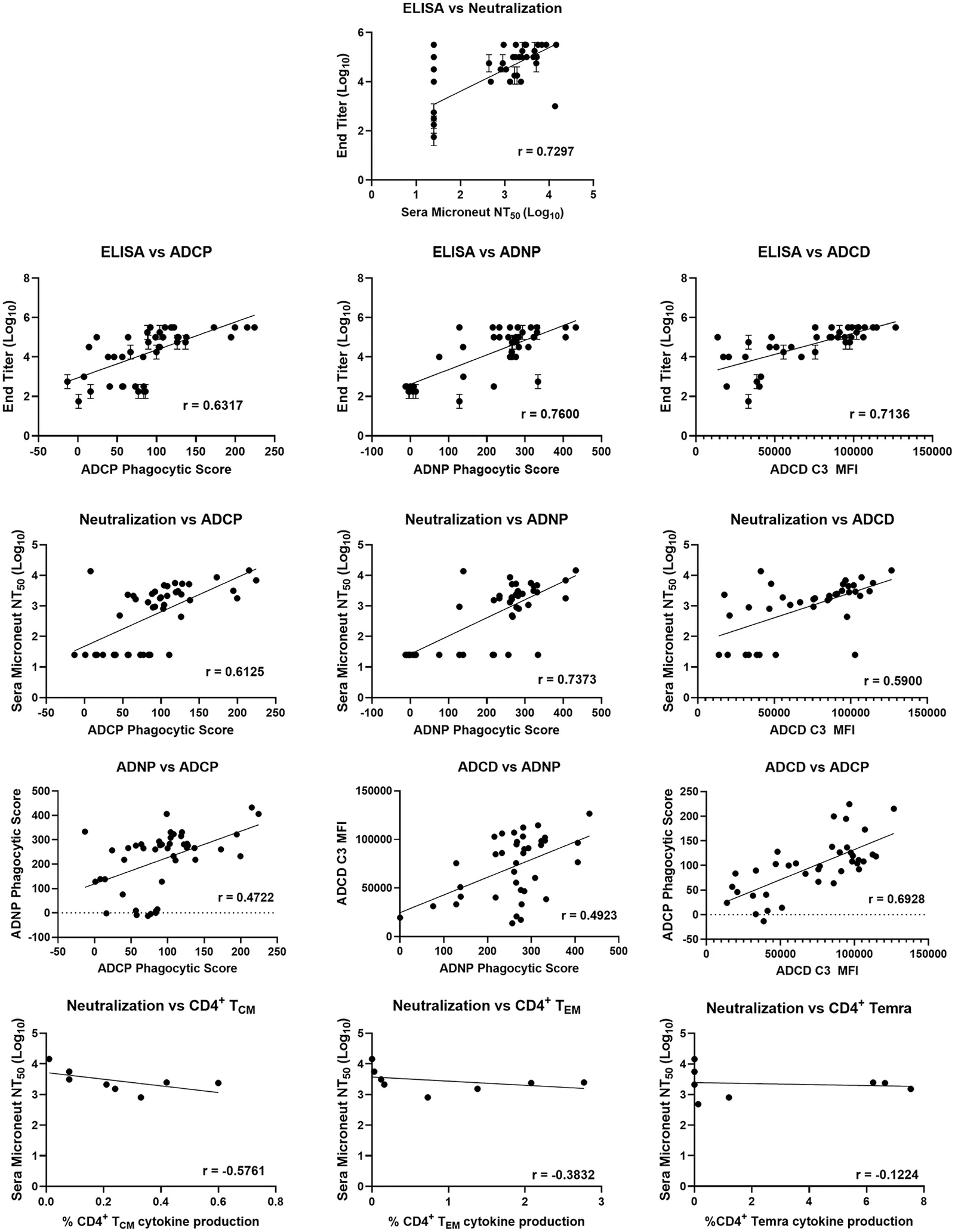
Correlation analysis between immunological readouts. Immunological parameters from VEEV ID-specific humoral assays were plotted to generate simple regression lines and the Pearson correlation coefficient (r) is reported.

We also evaluated correlations between various immunological readouts (i.e. neutralization activity and effector function), focusing on the VEEV ID-specific assays since the VEEV^+^ cohort was largest. As demonstrated in Fig 6, all humoral responses had fair to moderate correlation (as defined by Haldun Akoglu [54]), with r values ranging from 0.47 (ADNP vs ADCP, p < 0.01) to 0.76 (ELISA vs ADNP, p < 0.0001). Neutralization activity appears to have the strongest relationships with ELISA (r = 0.73, p < 0.0001) and ADNP (r = 0.74, p < 0.0001). Conversely, negative trends between ELISA and CD4^+^ T_CM_ cytokine production (r = -0.44, p = 0.27), and between neutralization and CD4^+^ T_CM_ cytokine production (r = -0.57, p = 0.13) were not powerful enough to demonstrate statistical significance, but limited conclusions can be drawn from these observations considering the smaller sample sizes of these assays. Cytokine production from additional T cell subsets demonstrated no correlation with humoral responses.

## Discussion

In this study we established a cohort of encephalitic alphavirus SPPs from Panama and developed a pipeline for evaluating correlates of immunological protection that can be leveraged to inform the rational design and evaluation of vaccines and therapeutics. Despite an increase in the incidence of cases, the risk of outbreaks associated with climate change and anthropogenic effects, and the pandemic potential of endemic encephalitic alphaviruses present in Central and South America, there is surprisingly little information about the human immune response to these viruses or how these responses correlate with protection or to animal modeling data. Here, we have demonstrated potent, broad and persistent humoral and cell-mediated responses in SPPs, illustrating the engagement of several aspects of the immune response that have been historically understudied in the context of these viruses.

While our SPP cohort has been previously described [19,21], we add to the previous findings by identifying significant ELISA cross-reactivity against multiple VEEV subtypes, as well as more heterologous MADV, WEEV, CHIKV and UNAV, particularly in our VEEV^+^ and DP cohorts. There have been previous reports of vaccine-associated cross-reactivity elicited between VEEV and arthritogenic alphaviruses [55,56], so the broad reactivity of VEEV^+^ and DP cohorts was not surprising. While broad cross-reactivity of mAbs isolated from EEEV survivors has also been demonstrated, these mAbs were directed against more cryptic and conserved E1-specific epitopes [39]. Thus, it is unlikely that we would replicate these findings due to 1) the heterogeneity of polyclonal sera, 2) the well-documented immunodominance of alphavirus E2-specific mAbs, and 3) the lack of recombinant antigen pre-treatment in our hands to expose cryptic E1 epitopes.

Neutralization of authentic virus represented a more difficult hurdle, but significant pan-VEEV cross-reactivity was demonstrated in VEEV^+^ participants, while MADV^+^ participants were also cross-reactive against EEEV. In our experience, the longevity of the neutralization response varies depending on the virus-specific cohort, but this study indicates persistent neutralizing antibody circulating >3 years post-alphavirus infection [45,46]. Moreover, in this study we report non-neutralizing functions of antibodies and the T-cell response for the first time from these VEEV+, MADV+ and DP participants.

Recent studies have demonstrated the importance of non-neutralizing activity associated with anti-VEEV and anti-MAYV mAb preclinical efficacy [28,57,58]. Here, we report rE1/E2-specific Fc effector function activity (ADCP, ADNP and ADCD) in human encephalitic alphavirus SPPs for the first time. We found marked ADNP activity (against both homologous and heterologous antigen) across cohorts and ADCP activity in VEEV^+^/DP participants. MADV^+^ individuals demonstrated less robust ADCP responses against homologous antigen (MADV), and no ADCP activity against heterologous antigen (VEEV IA/B and VEEV ID). Taken together, this suggests that the incorporation of Fc modifications to precisely target specific effector functions may benefit immunotherapeutic efficacy in the context of encephalitic alphaviruses [59–61].

In contrast to VEEV^+^ participants, 5/6 MADV^+^ mounted no detectable ADCD response against homologous antigen. In rodents, the lack of C3 (and thus complement cascade activation) induces severe encephalitic disease post-VEEV infection [62]. In humans, there is increased incidence of neurosequelae following MADV^+^ infection, and significantly more persistence in EEEV^+^ (compared to VEEV) individuals [22,24,44,63]. Although our sampling is small, it is interesting to speculate that there may be a link between ADCD activity and protection from neurological symptoms.

Currently, mechanistic differences between protective and pathogenic immune responses to encephalitic alphaviruses are poorly defined. In mice, the lack of B and T cells has been associated with increased survival and less disease post-VEEV infection, indicating an immunopathogenic component [64]. Conversely, antibody can mediate non-cytolytic alphavirus clearance from the CNS, and mice lacking B cells developed severe encephalitic disease following infection with an attenuated VEEV strain (V3533), only recovering following an influx of T cells and inflammatory monocytes, indicating a protective immune mechanism [65,66]. The distinctions between these seemingly contradictory findings have not yet been elucidated, highlighting the importance of validating preclinical findings in a consistent human cohort, and evaluating immune responses to correlate against clinical parameters.

To investigate T cell responses in a subset of our seropositive cohorts, we utilized an *ex vivo* stimulation assay on fresh PBMCs and on-site flow cytometry capabilities in Panama. We report for the first time VEEV- and MADV-specific polyfunctional cytokine (IFNy, IL-2) responses in CD4^+^ and CD8^+^ memory cell compartments (T_CM_, T_EM_ and T_EMRA_). Of the samples that were utilized for flow cytometry (9 VEEV^+^, 3 MADV^+^, 1 DP), several within the VEEV^+^ cohort (5/9) and the DP sample demonstrated potent (increased >3-fold compared to negative stimulation) cytokine production in response to VEEV and MADV rE1/E2. Cytokine responses within the MADV^+^ cohort were mostly negligible, although a single individual was reactive to MADV rE1/E2 within CD4^+^ and CD8^+^ T_CM_ compartments (Aza14). Given the small sample sizes and range of reactivity, demonstrating statistical significance within cohorts was quite difficult. However, future work will focus on investigating whether the strength of these responses correlates to disease severity, time post-infection or persistent sequelae with a larger sampling.

Clearly, the human convalescent immune response to encephalitic alphaviruses is complex. We did not find one dominant mechanism or pathway that obviously correlates with protection. Instead, some SPPs demonstrated potent B cell neutralization and effector function but were unresponsive in our T cell assay, or vice versa, while others demonstrated reactivity (or lack thereof) across compartments (S3 Fig). Within the B cell compartment, there were marked differences between strength of neutralization and effector function amongst individuals. Within the T cell compartment, rE1/E2 elicited cytokine production in a specific subset (i.e., T_CM_) in some, or across subsets in others.

Cohort demographic information and reported symptoms are described in S4 Fig. We also evaluated possible correlations between immunological readouts and frequency of overall reported symptoms, but saw no significant relationships (S5 Fig). It is possible that with a much larger sampling, more obvious trends may appear. While this work provided a wealth of new information, there were also some inherent limitations. Since this cohort was originally identified as part of a serosurveillance study, we were unable to correlate specific immune responses with exact time post-infection, severity of symptoms during the infection or recovery time. Now that we’ve demonstrated the utility of this analysis pipeline, future work will focus on increasing participant enrollment and addressing some of these questions. As an early, exploratory effort, our focus was on immunoreactivity to alphavirus glycoproteins due to the preponderance of evidence demonstrating induction of a potent anti-E1/E2 humoral response in the field [28–38]. To our knowledge the reactivity to more well-conserved viral proteins (i.e., nucleocapsid or NSPs) has not been explored in human alphavirus SPPs, but it’s interesting to speculate that leveraging these responses may induce more potent cross-protection. Finally, due to the expense of the stimulating antigens, as well as logistical limitations of performing these analyses in rural Panama, we were only able to perform flow cytometry assays on a subset of SPPs. However, we were able to assess cytokine responses in fresh, rather than frozen samples, circumventing any possible impacts cryopreservation may have on protein expression or cytokine production [67,68].

Working alongside regional scientists and experts in the field to assemble a sizable cohort of willing participants has provided the opportunity to thoroughly investigate the human immune response to encephalitic alphaviruses. Only by establishing and maintaining these collaborative pipelines can human healthcare benefit from studies of the long-lasting impacts of these viruses in endemic areas, particularly as vector habitats continue to expand due to environmental changes. Here, we’ve developed the logistical and methodological foundation for cohort evaluation, and demonstrated the wide variety and persistence of immunological pathways engaged by alphavirus exposure. Although much work remains to be done, our data indicates that VEEV infection was associated with greater cross-reactivity and should be utilized as a target to increase the likelihood of pan-encephalitic alphavirus breadth.

Until more studies are designed with a focus on global health to evaluate immune responses from acute phase through convalescence and correlate this heterogeneity with clinical outcomes, informed MCM development is difficult. This work demonstrates the variety, strength and persistence of the human encephalitic alphavirus response, and should be leveraged for the design of more balanced immunoregulatory and targeted vaccines and therapeutics.

## Supporting information

S1 Fig Sera neutralization against authentic alphaviruses. Sera samples were evaluated by microneutralization assay for reduction of Vero cell infection with VEEV subtypes (A) IA/B (Trinidad Donkey strain), (B) IC (P676 strain), (C) ID (3880 strain), (D) IE (Mena II strain), (E) MADV (Pan247188 strain), (F) EEEV (Fl93 strain), (G) WEEV (CBA87 strain), (H) CHIKV (AF15561 strain). Data represents dose response curves for each convalescent survivor, two replicate assays run in triplicate.
(JPG)

S2 Fig Representative scatter plot of cytokine production in CD8+ TCM cells post-stimulation from VEEV+ survivors. PBMCs were stimulated for 18 hours with VEEV or MADV rE1/E2, stained for dead cell exclusion, conventional T cell markers and intracellular cytokines, and analyzed by flow cytometry. CD8^+^CD45RA^-^CD95^+^CCR7^+^ antigen-specific T_CM_ from convalescent VEEV survivors produce cytokines (IFNy & IL-2) in response to VEEV and MADV rE1/E2 stimulation, while media only (negative control) samples are non-reactive.
(JPG)

S3 Fig Summary of immune responses in alphavirus SPPs. Relative strength of responsiveness by assay is reflected to differentiate trends in humoral, cell-mediated or overall immune activation following stimulation with alphavirus antigens. Blue represents a strong relative response, white a weak or non-existent relative response, and grey indicates a measurement not taken due to limited sample or antigen. Scale varies between assays.
(JPG)

S4 Fig Summary of participant demographics and reported symptoms post-infection. Responses were reported by participants as part of a questionnaire during serosurveillance collections. “% Reported Symptoms” is based on the total sequalae participants answered yes or no, symptoms with no response were discarded from the calculation.
(JPG)

S5 Fig Correlation between immune responses and frequency of reported symptoms. Responses were reported by participants as part of a questionnaire during serosurveillance collections. “% Reported Symptoms” is based on the total sequalae participants answered yes or no, symptoms with no response were discarded from the calculation. Immunological parameters from VEEV ID-specific humoral assays were plotted to generate simple regression lines and the Pearson correlation coefficient (r) is reported.
(JPG)
