# Supplementary material for: Encephalitic alphavirus infection induces long-term humoral and cell-mediated responses in seropositive participants from Panama
Source: PLoS Negl Trop Dis. 2026 Sep 11;20(9):e0014660. doi: 10.1371/journal.pntd.0014660 (PMC13592687; doi:10.1371/journal.pntd.0014660)
Supplement: Supplementary file 1 [file pntd.0014660.s006.docx]

De: **PLOS Pathogens** <[em@editorialmanager.com](mailto:em@editorialmanager.com)>
Date: lun, 11 ago 2025 a las 12:42
Subject: PLOS Pathogens Decision: PPATHOGENS-D-25-01604 - [EMID:80ce30a2440c6d05]
To: Sandra López-Vergés <[slopez@gorgas.gob.pa](mailto:slopez@gorgas.gob.pa)>

You are being carbon copied ("cc:'d") on an e-mail "To" "Andrew Herbert" [andrew.s.herbert4.civ@health.mil](mailto:andrew.s.herbert4.civ@health.mil)
CC: "Courtney A. Cohen" [courtney.a.cohen.civ@health.mil](mailto:courtney.a.cohen.civ@health.mil), "Jean Paul Carrera" [jpcarrera@gorgas.gob.pa](mailto:jpcarrera@gorgas.gob.pa), "Josefrancisco Galué" [josefrangalue@gmail.com](mailto:josefrangalue@gmail.com), "Davis Beltran" [dbeltran@gorgas.gob.pa](mailto:dbeltran@gorgas.gob.pa), "Rita Corrales" [rcorrales@gorgas.gob.pa](mailto:rcorrales@gorgas.gob.pa), "Luis Felipe Rivera" [lf_rivera@outlook.com](mailto:lf_rivera@outlook.com), "Zumara Chaverra" [zchaverra@gorgas.gob.pa](mailto:zchaverra@gorgas.gob.pa), "Yelissa Suarez" [yelissa_juarez@outlook.com](mailto:yelissa_juarez@outlook.com), "Jennifer Marciaga" [jmarciaga@gorgas.gob.pa](mailto:jmarciaga@gorgas.gob.pa), "Michelle L. Rock" [michellerock12@gmail.com](mailto:michellerock12@gmail.com), "Megan C. Ha" [megan.c.ha.ctr@health.mil](mailto:megan.c.ha.ctr@health.mil), "Darrell L. Wetzel" [darrell.l.wetzel.ctr@health.mil](mailto:darrell.l.wetzel.ctr@health.mil), "Dafna Abelson" [dafna.abelson@mappbio.com](mailto:dafna.abelson@mappbio.com), "Zachary A. Bornholdt" [zach.bornholdt@eitrbio.com](mailto:zach.bornholdt@eitrbio.com), "Crystal L. Moyer" [crystal.moyer@eitrbio.com](mailto:crystal.moyer@eitrbio.com), "Larry Zietlin" [larry.zeitlin@mappbio.com](mailto:larry.zeitlin@mappbio.com), "Juan M. Pascale" [jmpascale@gorgas.gob.pa](mailto:jmpascale@gorgas.gob.pa), "John Dye" [john.m.dye1.civ@health.mil](mailto:john.m.dye1.civ@health.mil), "Sandra López-Vergés" [slopez@gorgas.gob.pa](mailto:slopez@gorgas.gob.pa)

PPATHOGENS-D-25-01604
Encephalitic alphavirus infection induces humoral and cell-mediated responses in convalescent survivors from Panama.
PLOS Pathogens

Dear Dr. Herbert,

Thank you for submitting your manuscript to PLOS Pathogens. As with all papers, your manuscript was reviewed by members of the editorial board. Based on our assessment, we have decided that the work does not meet our criteria for publication and will therefore be rejected. If external reviews were secured, reviewers' comments will be included at the bottom of this email.

While we cannot consider your manuscript further for publication in PLOS Pathogens, we would like to offer you the option to transfer your submission to

| PLOS Neglected Tropical Diseases | <https://www.editorialmanager.com/PNTD/> |
| --- | --- |

.

Please note, all PLOS journals are editorially independent and vary in submission requirements.

Should you choose to transfer, your submission details and manuscript files will be transferred automatically, and you will receive a confirmation email within 24 hours.  Once transferred, your submission will be returned to you so you can check over your record before completing the submission. You may be asked to provide additional information. If you have any questions, please contact the editorial office of

| PLOS Neglected Tropical Diseases | <https://www.editorialmanager.com/PNTD/> |
| --- | --- |

. Please note that this offer will expire after 60 days from the decision date.

If you DO wish to transfer your submission, please click this link:

********

If you do NOT wish to transfer your submission, please click this link to decline:

********

Thank you once again for your support of PLOS and of open-access publishing. We hope that you will consider PLOS Pathogens for other submissions in the future.

Yours sincerely,

Deborah Lenschow
Academic Editor
PLOS Pathogens

Michael Letko
Section Editor
PLOS Pathogens

Michael Malim
Editor-in-Chief
PLOS Pathogens
[orcid.org/0000-0002-7699-2064Additional](http://orcid.org/0000-0002-7699-2064Additional) Editor Comments (if provided):

Thank you for the submission of your manuscript. It has been evaluated by 3 reviewers and while they all felt the data was interesting, there was concern that without the ability to relate the data to clinical disease manifestations and pathogenesis the study is only descriptive. There were also concerns raised that for the cross reactivity studies that without having data about exposure history tracked during the intervening time between the initial sero-survey and the follow-up study it is unclear if the results truly represent cross-reactivity of some level of new infection in the cohort.


[Note: HTML markup is below. Please do not edit.]

Reviewers' comments (if peer reviewed):

Reviewer's Responses to Questions

**Part I - Summary**
Please use this section to discuss strengths/weaknesses of study, novelty/significance, general execution and scholarship.

Reviewer #1: The manuscript by Cohen et al. analyzed antibody and T cell responses of survivors of Venezuelan equine encephalitis virus, Madariaga virus, or both. These studies fill key gaps in our understanding of the complicated correlates of immunity against these viruses, and highlight the importance of international collaboration in these studies. Some changes as listed below would improve the manuscript.

Reviewer #2: The manuscript evaluates the long-term humoral and T cell response from individuals previously infected with Venezuelan equine encephalitis virus (VEEV; subtype ID), Madariaga virus (MADV), or both, as determined from a serological survey. Samples were collected from survivors at a minimum of 3 years post-infection and analyzed for serum binding and neutralization of VEEV subtypes and related alphaviruses circulating in the area, Fc effector functions, and memory T cell responses. VEEV only and double positive patients developed broadly reactive antibody responses; however, cross-neutralization was more limited. Sera from MADV survivors showed reduced breadth of antibody binding. Furthermore, MADV immune serum only neutralized MADV. VEEV only and double positive serum samples were more potent inducers of ADCP, ADNP, and ADCD with VEEV antigens compared to MADV-immune serum. VEEV+, MADV+, and double positive sera samples all induced ADCP and ADNP with MADV antigens. Interestingly, MADV immune sera was a poor inducer of complement activity even when MADV antigens were used. VEEV-infected individuals showed a stronger CD4+ and CD8+ memory T cell response to both VEEV and MADV stimulation. For MADV only and double positive samples, only a potent CD8+ central memory T cell response was identified. These results showed differences between the adaptive immune response following infection with VEEV or MADV.
Overall, the manuscript provides a descriptive overview of the long-term adaptive immune response following VEEV and MADV infection. These results are useful and important findings for the field. However, due to the design of the study, the authors are unable to relate the results to clinical disease manifestation and pathogenesis, which limits the conclusions that can be drawn from the results.

Reviewer #3: The manuscript: Encephalitic alphavirus infection induces humoral and cell-mediated responses in convalescent survivors from Panama, by Cohen, et al., describes the characterization of post infection immune responses in a cohort of individuals previously exposed to either Venezuelan equine encephalitis virus (VEEV) and/or Madariaga virus (MADV). The studies characterize levels of virus specific and alphavirus-cross reactive binding and neutralizing antibody responses, Fc receptor dependent antibody effector functions, and virus specific T cell responses. The results indicate that VEEV convalescent individuals mount durable antibody responses that exhibits cross reactivity across VEEV strains, as well as MADV, CHIKV, and UNAV, while serum from MADV infected individuals showed less cross reactivity. Overall, the study provides interesting information on cross reactivity and functionality of serum from VEEV and/or MADV immune individuals. However, there are several concerns. The most notable weakness of the study is the lack of data on the nature of the disease, if any, suffered by the seropositive cohort. This makes is difficult to know whether differences in magnitude/functionality are related to disease severity or some other factor. It is also unclear whether the individuals’ exposure history was tracked during the intervening time between the initial sero-survey and the follow-up study. Therefore, it is unclear whether the cross reactivity across viruses seen in the study represents true cross reactivity, or some level of new infection within the cohort. Related to this last point, a more intensive analysis of the nature of the cross reactive immune response would significantly increase the impact of the study, such as characterizing whether there is specificity in the cross reactivity with specific proteins (E1, E2) or subdomains (e.g. E2 B domain).

**Part II – Major Issues: Key Experiments Required for Acceptance**
Please use this section to detail the key new experiments or modifications of existing experiments that should be absolutely required to validate study conclusions.

Generally, there should be no more than 3 such required experiments or major modifications for a "Major Revision" recommendation. If more than 3 experiments are necessary to validate the study conclusions, then you are encouraged to recommend "Reject".

Reviewer #1: 1) The authors should clearly state how these findings are different from those in references 21 and 52, where these donors were identified in the first place. It would be interesting to compare ELISA and PRNT results from these experiments and those in this manuscript.

We agree that it would be very interesting to compare assay outputs between institutes, and in fact this is within the scope of a future manuscript. Overall, the findings in the current manuscript are more focused on breadth of seroreactivity in this cohort with two future aims 1) to screen for monoclonal antibody discovery, and 2) to inform the rational design of future alphavirus-directed medical countermeasures. In contrast, refs 21 and 52 were limited to an evaluation of VEEV IAB (TrD) for PRNTs and recombinant VEEV IF for ELISAs for the purposes of critical serosurveillance in the region. We have added text in the discussion to reflect this, thank you for the suggestion.

2) The authors state “Conversely, MADV+ survivors were the only cohort able to significantly neutralize MADV (PAN247188 strain, p < 0.001) and EEEV (FL93 strain, p < 0.05), in contrast to the VEEV+ and DP survivors,” but Figure 2A shows that only VEEV+ survivors significantly neutralized EEEV.

Thank you for pointing this out, we have corrected our text accordingly.

Reviewer #2: 1. The authors should perform correlation analysis between the different immunological readouts to identify relationships, particularly between the binding antibodies, neutralizing antibodies, and Fc effector functions.

Thank you for the suggestion, we have performed these analyses, and updated the text/Figures accordingly.

2. Figure 2. What was used as the bottom of the curve for the neutralization area under the curve analysis? Some of the data presented in Supplemental Figure 1 are straight lines indicating no neutralization. Yet, most of the samples in Fig. 2A have AUC values greater than 10^4. It is unclear how samples with no detectable neutralization could have AUC values so high. Additionally, the 50% neutralization values should also be plotted. This should show greater differences between neutralizing and non-neutralizing samples.

We have replaced our AUC-style Figure 2 with NT50s (sera dilution that results in 50% neutralization of the virus in comparison to control-infected wells) based on common guidance from reviewers.

3. The authors mention that the study includes 47 enrollees; however, there are only 37 samples from infected individuals in the figures. Please clarify.

This is correct – our seropositive cohort currently has 47 enrollees. However, collections for this project have been occurring over several years, and only 37 were available during the snapshot of time we’re currently describing. We are preparing a subsequent manuscript to describe longitudinal responses, but felt it was too much information to compile into a single manuscript. However, to avoid future confusion, we wish to maintain the sample IDs (Aza1, Aza2, etc) that we use in the field, so we have attempted to clarify this in the Methods section.

4. Please include available demographics, such as sex and age for each group. Is there any indication that the participants were infected again during the post-infection 3 year interval?

Reviewer #3: The AUC presentation is not standard for neutralization assays, and data should also be presented in formats such as 50, 80, or 90% neutralizing titer (eg. NT50) or its equivalent.

We agree, and have updated Figure 2 accordingly.

2) It would be useful to know whether there are correlations between the different data types. For example, is there a correlation between ADCP, ADNP, or ADCD levels, and do any of these readouts correlate with neutralizing antibody levels for specific viruses?

Thank you for the suggestion. We have performed these analyses, and updated the text/Figures accordingly.

3) Information is needed on the limit of detection for each assay. For example, background neutralization levels are high in several of the assays, such as the MADV neut. assay (Fig 2A), which makes it difficult to interpret the results without clear indications of assay sensitivity. Given the high level of overlap between In Country Controls (ICC) and MADV+ group, it is somewhat surprising that these differences are significant, and it may be necessary to include additional controls (e.g. out of country controls) to allow the neutralization results for MADV to be interpreted.

**Part III – Minor Issues: Editorial and Data Presentation Modifications**
Please use this section for editorial suggestions as well as relatively minor modifications of existing data that would enhance clarity.

Reviewer #1: 1) Please show representative gating data for the Tcm identification for Figure S2 (FSC/SSC, doublets, live/dead, CD3/4/8, etc).

Reference has been added to the methods for gating strategy from our previous publication.

2) Figure 1 and 2 legend should list what the “+/+” samples are in Figures 1A and 2A.

Added, thank you.

3) Line 333, what is meant by “EEEV NA”?

“North American”, but now that MADV has been re-classified from South American EEEV, it is redundant, so it has been removed. Thank you.

4) The authors increase the font size of the % positive numbers in Q1, Q2, and Q3 in Supplemental Figure 2.

5) Supplemental Figure S3—although scale differs, a scale showing low (white) to high (various shades of blue) would be helpful.

Incorporated, thank you.

6) Figure 4D-F and 5D-F, a notation (asterisk?) on the scale bar would let the reader ascertain which individual had true positive T cell responses to each antigen.

We’re reluctant to define a level of “true” positive T cell response, but have indicated on the scale-bars the maximum baseline response following negative (media only) stimulation in these samples for clarity.

7) A phylogenetic tree comparing envelope sequences for the viruses in Figure 1 B would be helpful (and including EEEV as well)

8) A map of the collection sites in the country, and a table should be made listing information on enrollee location, positivity, sex, age, etc. would be useful.

Enrollees were all collected from a single village within the Darien province but we feel identifying it specifically on a map would violate participants’ privacy. We have incorporated a table with demographic information that is available in the Supplementary Material.

9) The authors should be commended for the very difficult T cell studies. Enhancing the methods for these assays would be useful to other investigators conducting similar studies. For example, what is the range of PMBC amounts/concentrations used? Was IL-2 added to the samples? Were 96 well round or flat-bottom plates used?

Thank you for the thoughtful comment. As indicated, performing such complex assays in a rural setting comes with a unique set of opportunities, but also challenges. As such, we aim to keep these assays as straight forward, and utilize as few reagents as possible. Thus, no IL-2 is incorporated. We do want to support other researchers in the field attempting similar crucial work, so we have attempted to enhance our Methods section for clarity. As indicated in the Methods section, we don’t like to standardize our PBMC input in the assay for several logistical considerations. First, due to both the time-cost during stimulation setup, which occurs immediately following a long day of collections in the field. More importantly, we prefer to maximize our yields rather than limit input to the lowest common denominator – we feel this is justified considering the memory cell types that we’re focused on are present at relatively low overall frequencies, it’s important to allow signal to rise above the assay noise as much as possible.

Reviewer #2: 1. In Figure 1, was the End Titer log transformed? If so, please adjust the y-axis on the graphs.

Incorporated, thank you.

2. In Fig S1, please color code the ICC, double positive, VEEV, and MADV groups so the different groups can be identified.

Incorporated.

3. The values in the key for Fig. 2B do not seem to match the values shown in Fig 2A. For example, the samples in the VEEV+ group all fall above 10^5 yet there is a gradient in the color in the heat map when the darkest blue is only at 8 x 10^4. Please check for accuracy.

The AUC figure was replaced.

4. In the heat maps for Fig 3, please indicate the groups with the lines as used in previous figures.

Incorporated, thank you.

5. For the T cell analysis with VEEV restimulation, please include which subtype of VEEV rE1/E2 was used for this analysis. Based on the data presented in Fig S3, it is assumed to be VEEV IAB. If so, please include rationale for using IAB rather than ID.

Incorporated into the Methods section.

6. In Fig S3, please keep the order of VEEV subtype consistent across each assay for ease of reading.

Reviewer #3: 1) Since there does not appear to be any disease data available on the donor populations, it is unclear what fraction of individuals were symptomatic or asymptomatic. Therefore, referring to individuals as survivors is somewhat extreme, and different terminology should be considered.

Thank you for the suggestion, we have adjusted our text accordingly.

2) Chikungunya is not a place name, and should not be capitalized when not leading a sentence.

Edited, thank you.
