# Supplementary material for: Encephalitic alphavirus infection induces long-term humoral and cell-mediated responses in seropositive participants from Panama
Source: PLoS Negl Trop Dis. 2026 Sep 11;20(9):e0014660. doi: 10.1371/journal.pntd.0014660 (PMC13592687; doi:10.1371/journal.pntd.0014660)
Supplement: Supplementary file 2 [file pntd.0014660.s007.docx]

**Response to Reviewers:**

METHODS:

Line 130 – Mention the availability of the cohort demographic information and reported symptoms that are shown in Supplemental Figure 4 in this section rather only mentioning it, in line 379 which is part of the discussion.

This has been incorporated, thank you for the suggestion.

Line 151 – Please state strains used to construct recombinant proteins such as the strains mentioned in line 172-174.

This has been incorporated, thank you for the suggestion.

Line 151 - VEEV IE is not listed, however; Figure 1 shows VEEV IE rE1/E2, please clarify.

This was an oversight, and has been incorporated, thank you for catching.

Line 151 – Inconsistent writing of VEEV IA/B in the manuscript.

This has been corrected, thank you for catching.

RESULTS:

Figure 1 - Significance brackets are depicted inconsistently across figures. For example, in Figure 1 some panels include brackets for non-significant comparisons (e.g., VEEV IE) while others omit them such as in the case of MADV. Please update Figures 1, 2, and 3 so that brackets are shown consistently for all comparisons. If only selected pairwise comparisons were tested, please state this explicitly in the figure legends.

This has been corrected, thank you for catching. We’ve removed any reference to “ns” in these figures, and added an explanation of pairwise statistics in the figure legends.

Figure 4 and 5 – Figures 4 and 5 show inconsistent y-axis numbering around the axis break. In Figure 4, panel A is missing the value at the lowest tick mark, making it unclear where the break begins. This is handled correctly in panels B and C, where the lowest tick is labeled. However, the issue is most pronounced in Figure 5, where most data points fall between an unlabeled tick mark and the nearest labeled one, making the values difficult to read. Please ensure y-axis tick marks are consistently labeled across all panels, particularly around axis breaks.

This has been corrected, thank you for catching.

REVIEWER #1 MINOR COMMENTS:
The abstract indicates that the cohort contains 47 individuals but the analysis only included 37. Please make this consistent throughout.

This has been corrected, thank you for catching.

Figure 1 – Can the authors comment on the convalescent sera from VEEV+ individuals having broad reactivity against all alphavirus E1/E2 proteins tested, as opposed to MADV which had a more limited reactivity? How does this correlate with conservation of the E1/E2 glycoproteins across these viruses?

Thank you for the comment, we have expanded our discussion around this data to address this question.

Supplementary Figure 5 – can the authors look at a correlation analysis of immune response vs a subset of the reported symptoms, such as just with the neurological symptoms (e.g. memory loss, headache, etc.)?

Thank you for the suggestion – we ran analyses between the most prevalent symptom reported (“headache”) and several humoral responses (ELISA, neutralization and ADNP), and were not able to determine any correlations between these parameters. We think that given the variability in long-term sequelae between seropositive individuals, the size of the cohort will need to be expanded significantly before this type of analysis is powerful enough to identify trends. However, we agree that this would be very interesting and impactful, and it is a long-term goal of the collaboration.

Line 332-334 – no correlation plots for the T-cell response are shown within the manuscript. Please provide these in the supplemental figures.

These have been added to Fig 6.
