# Supplementary material for: Encephalitic alphavirus infection induces long-term humoral and cell-mediated responses in seropositive participants from Panama
Source: PLoS Negl Trop Dis. 2026 Sep 11;20(9):e0014660. doi: 10.1371/journal.pntd.0014660 (PMC13592687; doi:10.1371/journal.pntd.0014660)
Supplement: Peer review history

# Author comment: Author response to Decision Letter 0

**DOI:** 10.1371/journal.pntd.0014660.r001

**Transfer Alert**

This paper was transferred from another journal. As a result, its full editorial history (including decision letters, peer reviews and author responses) may not be present.

17 Dec 2025

Attachment Submitted filename: PLOS Path Response to Reviewers.docx

# Peer review: Decision Letter 0

**DOI:** 10.1371/journal.pntd.0014660.r002

**Authors:** Sujatha Sunil, Colleen Jonsson

1 Apr 2026

Response to Reviewers Revised Manuscript with Track Changes Manuscript

Shaden Kamhawi

co-Editor-in-Chief

PLOS Neglected Tropical Diseases

orcid.org/0000-0003-4304-636XX

Paul Brindley

co-Editor-in-Chief

PLOS Neglected Tropical Diseases

orcid.org/0000-0003-1765-0002

**Journal Requirements:**

1) Please ensure that the CRediT author contributions listed for every co-author are completed accurately and in full.

At this stage, the following Authors/Authors require contributions: Courtney A. Cohen, Jean Paul Carrera, Josefrancisco Galué, Davis Beltran, Rita Corrales, Luis Felipe Rivera, Zumara Chaverra, Yelissa Suarez, Jennifer Marciaga, Michelle L. Rock, Megan C. Ha, Darrell L. Wetzel, Dafna Abelson, Zachary A. Bornholdt, Crystal L. Moyer, Larry Zietlin, Juan M. Pascale, John Dye, Sandra López-Vergés, and Andrew Herbert. Please ensure that the full contributions of each author are acknowledged in the "Add/Edit/Remove Authors" section of our submission form.

The list of CRediT author contributions may be found here: https://journals.plos.org/plosntds/s/authorship#loc-author-contributions

2) Please upload all main figures as separate Figure files in .tif or .eps format. For more information about how to convert and format your figure files please see our guidelines:

https://journals.plos.org/plosntds/s/figures

3) We notice that your supplementary Figures are included in the manuscript file. Please remove them and upload them with the file type 'Supporting Information'. Please ensure that each Supporting Information file has a legend listed in the manuscript after the references list.

**Reviewers' comments:**

**Key Review Criteria Required for Acceptance?**

As you describe the new analyses required for acceptance, please consider the following:

**Methods:**

-Are the objectives of the study clearly articulated with a clear testable hypothesis stated?

-Is the study design appropriate to address the stated objectives?

-Is the population clearly described and appropriate for the hypothesis being tested?

-Is the sample size sufficient to ensure adequate power to address the hypothesis being tested?

-Were correct statistical analysis used to support conclusions?

-Are there concerns about ethical or regulatory requirements being met?

Reviewer #1: See comments below

Reviewer #2: Minor revision please find comments below

Line 130 – Mention the availability of the cohort demographic information and reported symptoms that are shown in Supplemental Figure 4 in this section rather only mentioning it, in line 379 which is part of the discussion.

Line 151 – Please state strains used to construct recombinant proteins such as the strains mentioned in line 172-174.

Line 151 - VEEV IE is not listed, however; Figure 1 shows VEEV IE rE1/E2, please clarify.

Line 151 – Inconsistent writing of VEEV IA/B in the manuscript.

**********

**Results:**

-Does the analysis presented match the analysis plan?

-Are the results clearly and completely presented?

-Are the figures (Tables, Images) of sufficient quality for clarity?

Reviewer #1: See comments below

Reviewer #2: Minor revision please find comments below

Figure 1 - Significance brackets are depicted inconsistently across figures. For example, in Figure 1 some panels include brackets for non-significant comparisons (e.g., VEEV IE) while others omit them such as in the case of MADV. Please update Figures 1, 2, and 3 so that brackets are shown consistently for all comparisons. If only selected pairwise comparisons were tested, please state this explicitly in the figure legends.

Figure 4 and 5 – Figures 4 and 5 show inconsistent y-axis numbering around the axis break. In Figure 4, panel A is missing the value at the lowest tick mark, making it unclear where the break begins. This is handled correctly in panels B and C, where the lowest tick is labeled. However, the issue is most pronounced in Figure 5, where most data points fall between an unlabeled tick mark and the nearest labeled one, making the values difficult to read. Please ensure y-axis tick marks are consistently labeled across all panels, particularly around axis breaks.

**********

**Conclusions:**

-Are the conclusions supported by the data presented?

-Are the limitations of analysis clearly described?

-Do the authors discuss how these data can be helpful to advance our understanding of the topic under study?

-Is public health relevance addressed?

Reviewer #1: See comments below

Reviewer #2: The conclusions are supported by the findings, and the authors acknowledge the limitations of their results and how these constraints affect the interpretation of their data.

**********

**Editorial and Data Presentation Modifications?**

Use this section for editorial suggestions as well as relatively minor modifications of existing data that would enhance clarity. If the only modifications needed are minor and/or editorial, you may wish to recommend “Minor Revision” or “Accept”.

Reviewer #1: See comments below

Reviewer #2: Minor revisions, please refer to comments in results section.

**********

**Summary and General Comments**

Use this section to provide overall comments, discuss strengths/weaknesses of the study, novelty, significance, general execution and scholarship. You may also include additional comments for the author, including concerns about dual publication, research ethics, or publication ethics. If requesting major revision, please articulate the new experiments that are needed.

Reviewer #1: This study provides a detailed characterization of long-term immune responses in individuals seropositive for Venezuelan equine encephalitis virus (VEEV) or Madariaga virus (MADV). The patient cohort was from the Darién region of Panama which has a history of alphavirus infections. Both humoral and cell-mediated immune responses were assessed in this patient cohort. Overall the VEEV positive patients had more robust and broader humoral responses as compared to MADV positive patients. This study is the first to determine the levels of antibody-dependent cellular phagocytosis (ADCP), antibody-dependent, neutrophil phagocytosis (ADNP), and antibody-dependent complement deposition (ADCD) in VEEV or MADV patient sera. Unfortunately correlation analysis found no correlation between the immune responses and patient symptoms. Nonetheless, this is an important study to provide fundamental information on long-term immune responses following encephalitic alphavirus infection in humans.

Minor comments

The abstract indicates that the cohort contains 47 individuals but the analysis only included 37. Please make this consistent throughout.

Figure 1 – Can the authors comment on the convalescent sera from VEEV+ individuals having broad reactivity against all alphavirus E1/E2 proteins tested, as opposed to MADV which had a more limited reactivity? How does this correlate with conservation of the E1/E2 glycoproteins across these viruses?

Supplementary Figure 5 – can the authors look at a correlation analysis of immune response vs a subset of the reported symptoms, such as just with the neurological symptoms (e.g. memory loss, headache, etc.)?

Line 332-334 – no correlation plots for the T-cell response are shown within the manuscript. Please provide these in the supplemental figures.

Reviewer #2: The authors studied the humoral and cell-mediated responses from a cohort of individuals previously shown to be seropositive for New World alphaviruses, VEEV and/or MADV. Samples were collected 3 years post infection. Collected samples were analyzed to determine cross-reactivity to multiple VEEV and MADV subtypes, Fc effector functions and T cell responses. Convalescent sera from VEEV+ and double positive (DP) individuals showed broad activity against VEEV subtypes, whereas MADV sera showed limited cross-reactivity. Similar trends were observed for ADCP, ADNP, and ADCD activity, where VEEV and DP sera resulted in more broad activation compared with MADV. Lastly, it was shown VEEV+ individuals produced more potent cytokine responses and their activity was driven by broader T cell cross-activity, compared with MADV+ individual.

**********

PLOS authors have the option to publish the peer review history of their article (what does this mean?). If published, this will include your full peer review and any attached files.

If you choose “no”, your identity will remain anonymous but your review may still be made public.

**Do you want your identity to be public for this peer review?**  For information about this choice, including consent withdrawal, please see our Privacy Policy.

Reviewer #1: No

Reviewer #2: No

**Figure resubmission:**

**Reproducibility:** -->To enhance the reproducibility of your results, we recommend that authors of applicable studies deposit laboratory protocols in protocols.io, where a protocol can be assigned its own identifier (DOI) such that it can be cited independently in the future. Additionally, PLOS ONE offers an option to publish peer-reviewed clinical study protocols. Read more information on sharing protocols at https://plos.org/protocols?utm_medium=editorial-email&utm_source=authorletters&utm_campaign=protocols-->?>

# Author comment: Author response to Decision Letter 1

**DOI:** 10.1371/journal.pntd.0014660.r003

10 Jun 2026

Attachment Submitted filename: Response to PNTD Reviewers_final.docx

# Peer review: Decision Letter 1

**DOI:** 10.1371/journal.pntd.0014660.r004

**Authors:** Sujatha Sunil, Colleen Jonsson

10 Aug 2026

Dear Dr. Herbert,

We are pleased to inform you that your manuscript 'Encephalitic alphavirus infection induces long-term humoral and cell-mediated responses in seropositive participants from Panama.' has been provisionally accepted for publication in PLOS Neglected Tropical Diseases.

Before your manuscript can be formally accepted you will need to complete some formatting changes, which you will receive in a follow up email. A member of our team will be in touch with a set of requests.

Please note that your manuscript will not be scheduled for publication until you have made the required changes, so a swift response is appreciated.

IMPORTANT: The editorial review process is now complete. PLOS will only permit corrections to spelling, formatting or significant scientific errors from this point onwards. Requests for major changes, or any which affect the scientific understanding of your work, will cause delays to the publication date of your manuscript.

Should you, your institution's press office or the journal office choose to press release your paper, you will automatically be opted out of early publication. We ask that you notify us now if you or your institution is planning to press release the article. All press must be co-ordinated with PLOS.

Thank you again for supporting Open Access publishing; we are looking forward to publishing your work in PLOS Neglected Tropical Diseases.

Best regards,

Colleen B Jonsson, PhD

Academic Editor

PLOS Neglected Tropical Diseases

Sujatha Sunil

Section Editor

PLOS Neglected Tropical Diseases

Shaden Kamhawi

co-Editor-in-Chief

PLOS Neglected Tropical Diseases

orcid.org/0000-0003-4304-636XX

Paul Brindley

co-Editor-in-Chief

PLOS Neglected Tropical Diseases

orcid.org/0000-0003-1765-0002

***********************************************************

p.p1 {margin: 0.0px 0.0px 0.0px 0.0px; line-height: 16.0px; font: 14.0px Arial; color: #323333; -webkit-text-stroke: #323333}span.s1 {font-kerning: none

Reviewer's Responses to Questions

**Key Review Criteria Required for Acceptance?**

As you describe the new analyses required for acceptance, please consider the following:

**Methods**

-Are the objectives of the study clearly articulated with a clear testable hypothesis stated?

-Is the study design appropriate to address the stated objectives?

-Is the population clearly described and appropriate for the hypothesis being tested?

-Is the sample size sufficient to ensure adequate power to address the hypothesis being tested?

-Were correct statistical analysis used to support conclusions?

-Are there concerns about ethical or regulatory requirements being met?

Reviewer #1: (No Response)

Reviewer #2: (No Response)

**********

**Results**

-Does the analysis presented match the analysis plan?

-Are the results clearly and completely presented?

-Are the figures (Tables, Images) of sufficient quality for clarity?

Reviewer #1: (No Response)

Reviewer #2: Figure 2 panel A is missing.

**********

**Conclusions**

-Are the conclusions supported by the data presented?

-Are the limitations of analysis clearly described?

-Do the authors discuss how these data can be helpful to advance our understanding of the topic under study?

-Is public health relevance addressed?

Reviewer #1: (No Response)

Reviewer #2: (No Response)

**********

**Editorial and Data Presentation Modifications?**

Use this section for editorial suggestions as well as relatively minor modifications of existing data that would enhance clarity. If the only modifications needed are minor and/or editorial, you may wish to recommend “Minor Revision” or “Accept”.

Reviewer #1: (No Response)

Reviewer #2: Figure 2 panel A is missing and previous submissions figure was used for current evaluation.

**********

**Summary and General Comments**

Use this section to provide overall comments, discuss strengths/weaknesses of the study, novelty, significance, general execution and scholarship. You may also include additional comments for the author, including concerns about dual publication, research ethics, or publication ethics. If requesting major revision, please articulate the new experiments that are needed.

Reviewer #1: The authors have addressed the prior critiques.

Reviewer #2: (No Response)

**********

PLOS authors have the option to publish the peer review history of their article (what does this mean?). If published, this will include your full peer review and any attached files.

If you choose “no”, your identity will remain anonymous but your review may still be made public.

**Do you want your identity to be public for this peer review?**  For information about this choice, including consent withdrawal, please see our Privacy Policy.

Reviewer #1: No

Reviewer #2: No

# Editor report: Acceptance letter

**DOI:** 10.1371/journal.pntd.0014660.r005

**Authors:** Sujatha Sunil, Colleen Jonsson

Dear Dr. Herbert,

We are delighted to inform you that your manuscript, "Encephalitic alphavirus infection induces long-term humoral and cell-mediated responses in seropositive participants from Panama.," has been formally accepted for publication in PLOS Neglected Tropical Diseases.

We have now passed your article onto the PLOS Production Department who will complete the rest of the publication process. All authors will receive a confirmation email upon publication.

The corresponding author will soon be receiving a typeset proof for review, to ensure errors have not been introduced during production. Please review the PDF proof of your manuscript carefully, as this is the last chance to correct any scientific or type-setting errors. Please note that major changes, or those which affect the scientific understanding of the work, will likely cause delays to the publication date of your manuscript. Note: Proofs for Front Matter articles (Editorial, Viewpoint, Symposium, Review, etc...) are generated on a different schedule and may not be made available as quickly.

Soon after your final files are uploaded, the early version of your manuscript will be published online unless you opted out of this process. The date of the early version will be your article's publication date. The final article will be published to the same URL, and all versions of the paper will be accessible to readers.

For Research Articles, you will receive an invoice from PLOS for your publication fee after your manuscript has reached the completed accept phase. If you receive an email requesting payment before acceptance or for any other service, this may be a phishing scheme. Learn how to identify phishing emails and protect your accounts at https://explore.plos.org/phishing.

Thank you again for supporting open-access publishing; we are looking forward to publishing your work in PLOS Neglected Tropical Diseases.

Best regards,

Shaden Kamhawi

co-Editor-in-Chief

PLOS Neglected Tropical Diseases

Paul Brindley

co-Editor-in-Chief

PLOS Neglected Tropical Diseases
